# Colonisation in social species: the importance of breeding experience for dispersal in overcoming information barriers

**DOI:** 10.1038/srep42866

**Published:** 2017-02-17

**Authors:** A. Payo-Payo, M. Genovart, A. Sanz-Aguilar, J. L. Greño, M. García-Tarrasón, A. Bertolero, J. Piccardo, D. Oro

**Affiliations:** 1 GEP, IMEDEA (CSIC-UIB), Esporles, Spain; 2 CEAB (CSIC), Blanes, Spain; 3 Ecology Area, Department of Applied Biology, Miguel Hernández University, Elche, Spain; 4 GOU, Castellón, Spain; 5 Department Biologia Animal, Facultat de Biologia, Universitat de Barcelona, Spain; 6 Associació Ornitològica Picampall de les Terres de l’Ebre, Amposta, Spain

## Abstract

Studying colonisation is crucial to understand metapopulations, evolutionary ecology and species resilience to global change. Unfortunately, few empirical data are available because field monitoring that includes empty patches at large spatiotemporal scales is required. We examine the colonisation dynamics of a long-lived seabird over 34 years in the western Mediterranean by comparing population and individual data from both source colony and the newly-formed colonies. Since social information is not available, we hypothesize that colonisation should follow particular dispersal dynamics and personal information must be crucial in decision making. We test if adverse breeding conditions trigger colonisation events, if personal information plays a role in colonisation and if colonisers experience greater fitness. Our results show a temporal mismatch between colonisation events and both density-dependence and perturbations at the source colony, probably because colonisers needed a longer prospecting period to compensate for the lack of public information. Colonisers were mostly experienced individuals gaining higher breeding success in the new colony. Our results highlight the demographic value that experienced individuals can have on metapopulation dynamics of social long-lived organisms.

Species persistence in a changing world depends on the ability to respond to environmental changes^1^,^2^. A mechanism by which species can cope with such changes is by modifying their spatio-temporal distribution and colonising new environments^2^. Unfortunately, the empirical study of colonisation has been largely anecdotal — mostly in non-social species and through theoretical models^3^,^4^. Colonisations occur at large spatio-temporal scales and are seldom documented, particularly in long-lived vertebrates^5^,^6^. Therefore, more information on colonisation processes (e.g., drivers and fitness consequences) and the individual characteristics of colonisers (e.g., experience or age)^5^,^7^ is required.

Dispersal is a multi-step decision process: Individuals must first decide to disperse from their natal or breeding colony; then, individuals must decide between settling in an already occupied site or colonising a new empty patch^8^,^9^. These decisions require reliable information—anything reducing uncertainty—about the alternatives^10^. Such decisions are more likely to lead to positive outcomes if based on information, reducing uncertainty about the suitability of alternatives^10^. Colonisation is thus a risky endeavour since social information may not be available (or only available through heterospecific habitat copying) and individuals must rely solely on personal information.

Until the 2000’s the Audouin’s gull (*Ichthyaetus audouinii*) colony at Punta de la Banya (hereafter, source colony) was the most important breeding site hosting up to 70% of the species’ world population^9^. During the last three decades, around 20 new Audouin’s gull colonies formed along the western Mediterranean providing us with a unique dataset to study patch colonisation. We address three specific questions (1) Do adverse breeding conditions trigger colonisation? (2) Does personal information play an important role? (3) Do colonisers experience greater fitness? We hypothesize that personal information is relevant. We expect to find: (a) a temporal delay between perturbations triggering dispersal and colonisation (i.e., an amount of time required to explore empty patches and to gather information), (b) that colonisers should be experienced breeders (i.e., previous breeding experience might reduce uncertainty), and (c), since choosing a breeding patch is a risky decision, colonisation should lead to increased population fitness (i.e., higher breeding success)^11^.

We searched for qualitative association with adverse breeding conditions by identifying breaking points on the temporal distribution of colonisations. Then, we tested the role of experience in one of three different ways: Firstly, we tested for differences in the proportions of inexperienced and experienced breeders present at new colonies and the source colony. Secondly, we compared the proportion of successful and unsuccessful breeders (BSq_t–1_) that decided to return and breed again at the source colony or to settle in a new colony. Thirdly, we tested for differences in the laying date (LD), clutch size (CS) and egg volume (V) at the source colony in the year preceding colonisation (t-1) between newly colonizing individuals and philopatric birds (t). Finally, we compared breeding success of a new colony (La Ràpita Port) with the source colony, and specifically with two sub-colonies (i.e., patches) within the source colony with similar age distribution but differing in habitat features related to accessibility for predators.

## Results

**Figure 1:**
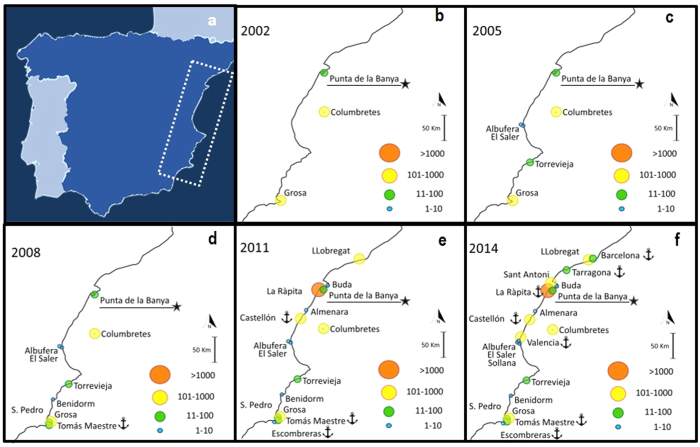
Panel (a) Iberian Peninsula and study area along the western Mediterranean coast (surrounded by white dashed line). We did not include colonies from the Balearic Islands since they have little exchange of individuals with the mainland system^9^. Panels (b) to (f) show the temporal evolution of Audouin’s gull colonisation events at regular time intervals from 1975. Circle size is proportional to the number of colonisers in the year of colony foundation; anchors indicate colonies settled in port areas (see Table 1 for colony details). A video with a complete temporal evolution of colonizations is in Appendix S1. Punta de la Banya is the source colony (underlined and marked with a star)^9^. Maps were built in R-Software^38^.

**Figure 2:**
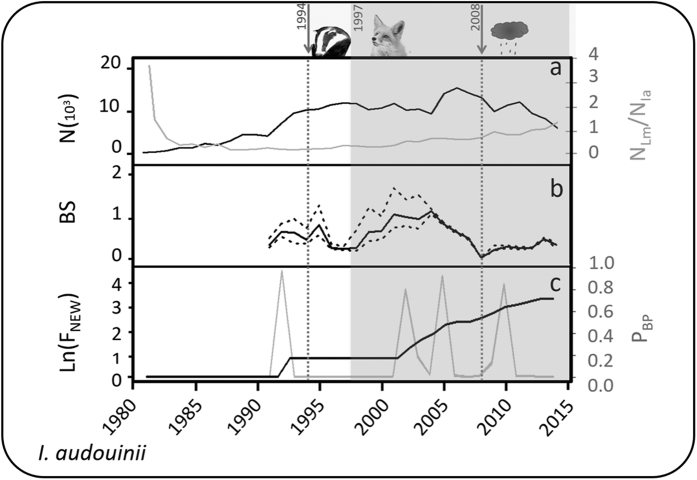
Occurrence of perturbations at *Punta de la Banya* since colonisation (1981–2015): arrows show punctual presence of a badger and an extreme cold storm in different years; shadowed area denotes continuous presence of foxes; Panel (a) Audouin’s gull population density fluctuations (black solid line, number of pairs N * 10^3^); N_Lm_/N_Ia_, ratio of population size between Yellow-legged and Audouin’s gulls (grey solid line). Panel b) BS, Audouin’s gull breeding success at the *Punta de la Banya* colony (number of fledglings/pair, (95%CI)). Panel (c) Ln (F_NEW_), neperian logarithm of the accumulated frequency of new colonies by year in the Western Mediterranean (black solid line) and P_BP_, probability of breaking points for the Ln (F_NEW_) temporal series (grey solid line). Fox and Badger images were modified from the Flickr photos “redfox10” and “Badger” which are copyright (**c**) 2011 Peggy cardigan https://flic.kr/p/kFvEbZ and (**c**) 2012 Peter Trimming https://flic.kr/p/d5CkEJ respectively. Both images can be used under a CC by 2.0 https://creativecommons.org/licenses/by/2.0/.

Since 1981, and particularly since 2003, 19 new colonies were established along the western Mediterranean (Table 1, Figs 1 and 2, see a video of colonisation process in Supplementary Material Appendix 1). We detected high probabilities of breaking points—points that divide data series into blocks such that the mean is constant within each block — in 1992, 2003, 2005 and 2010 (Fig. 2c). This analysis identified three major phases: (a) a 1992 breaking point corresponded with the formation of Grosa colony, (b) colonisation events in 2003 and 2005 coincided with colonisations of six mostly natural sites in the southern part of the study area, and (c) the colonisation of port sites in 2010 (Fig. 1). 37% of the new colonies appeared inside or near ports (Fig. 1). There was no obvious linkage of colonisation events to adverse breeding conditions in the previous breeding seasons (Fig. 2).

In the first year of colonisation, new colonies hosted higher proportions of experienced individuals than the source colony (87.1% and 63.5%, respectively, *χ*^2^ = 176.895, df = 1, p < 0.0001). There was no difference in the proportion of previously successful breeders between the new and the source colony in the year of colonisation (BSq_t-1_, *χ*^2^ = 0.739, df = 1, p = 0.390). In other words, settlement colony (source vs. new) was not related to the breeding success experienced by individuals the previous year (see electronic Supplementary Material S2, Table S2). Colonisers and philopatric individuals also showed similar egg volume, clutch size and laying date in the year before colonisation (see electronic Supplementary Material S2, Table S3).

**Figure 3:**
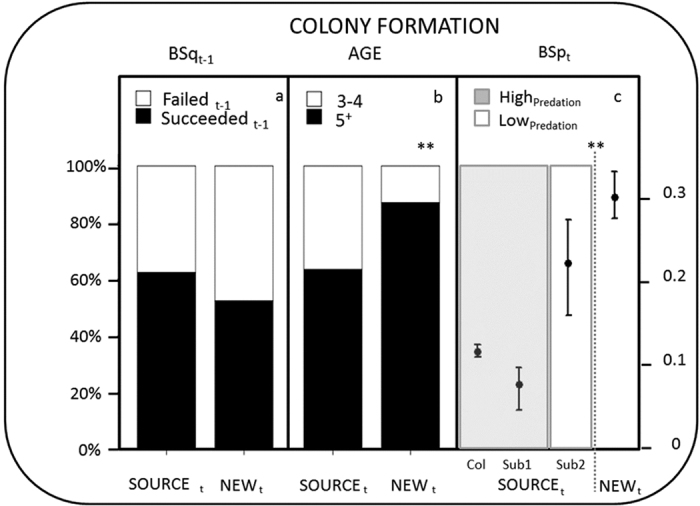
Colony formation patterns for Audouin’s gull breeding in the Western Mediterranean. SOURCE refers to individuals present at the Punta de la Banya colony and NEW to individuals present at new formation colonies. Panel (a) represents proportion of individuals present in SOURCE colony and NEW colonies at time t, conditional to their breeding success at t-1 (failed vs succeeded). Panel (b) represents distribution of individuals by means of breeding experience: inexperienced 3–4 years (white) vs experienced 5 years or more (black). Panel (c) represents breeding success of SOURCE colony and NEW colonies at time t with high (grey shadow) and low (no shadow) predation levels. Notice that SOURCE colony includes the average breeding success of individuals breeding at different sub-colonies (patches) at the source colony (Col), and Sub1 and Sub2 are the two sub-colonies within the SOURCE colony with similar age structure to the NEW colony (see Methods) (Sub1, Miseria and Sub2, Alfacs. **p < 0.001 significance level).

In the year of its colonisation (2011), breeding success (BSp_t_) was higher at the new colony (La Ràpita Port), BSp_t_ = 0.31(95% CI, 0.29–0.34), than at the source colony, BSp_t_ = 0.14 (95% CI, 0.13–0.15), and at both source sub-colonies with a similar age structure to the new colony, BSp_tsub1_ = 0.24 (95% CIs, 0.18–0.29) vs BSp_tsub2_ = 0.1(95% CIs, 0.07–0.12) (Fig. 3). In 2011, the new colony was free of predation, as was one sub-colony at the source which was surrounded by water (Sub_1_), preventing access by terrestrial predators. The other sub-colony (Sub_2_), as with many sites at the source colony, suffered from intense carnivore disturbance and predation. Colonies that were more accessible to terrestrial predators experienced lower breeding success (Fig. 3).

## Discussion

Colonisation is a crucial process in metapopulation dynamics and may be critical when assessing a species’ ability to respond to perturbations^1^,^2^. Its drivers, the characteristics of colonisers and a quantification of its pay-offs remain fairly unexplored^5^,^6^,^11^. Our results show that colonisations seem temporally and spatially unpredictable and they occur in response to an accumulation of perturbations exceeding an unknown threshold. Compared to source colonies, a disproportionate number of colonising individuals were older birds with greater breeding experience. In turn these birds had higher breeding success at newly established breeding colonies.

The temporal mismatch between adverse breeding conditions and colonisations may be in response to several processes which might not be mutually exclusive. First, individuals may face a trade-off between being philopatric, thus taking advantage of their previous experience and colonising a new patch without experience or social information available^10^. At the source colony, higher occupation of sites surrounded by water was likely a behavioural resilience mechanism to mitigate the effects of predation and to avoid the inherent risks of colonisation^12^. This behavioural resilience may delay colonisations of new patches, but it probably has a threshold, particularly when perturbations are consecutive^13^,^14^. Second, several studies have recorded some individuals visiting empty patches years before breeding, probably to collect information about habitat suitability^10^,^15^,^16^,^17^. Resilience and philopatry may thus delay the appearance of tipping points in colonisation, and may result in a non-linear relationship between adverse breeding conditions and colonisation^18^.

The use of non-natural environments (i.e., ports) appeared as a cultural innovation for the species in the study area, in a similar manner to that which occurred in a naval port in Corsica in 1990^19^. This innovation suggests adaptation to novel environments, and the spread of port colonisations in recent years suggests that colonisers rely on experience and obtain information from already occupied patches to reduce uncertainty^10^.

Previous studies at the source colony showed that predation caused partial breeding failure and immediate high dispersal to already occupied patches^13^. Nevertheless, colonisations did not occur immediately after deterioration of breeding conditions. Colonisation seems to follow special dispersal dynamics. Colonisers were experienced individuals that may be followed by young and inexperienced individuals in subsequent years, once the patch is occupied^6^,^9^,^16^,^20^.

Colonisation should only occur when advantages of colonisation outweigh its risks^11^. We detected higher breeding performance at the new colony probably due to lower predation risks. Increased fitness should be expected following successful colonisations (i.e., colonies persisting over time); however, that might not always be the case since, given the lack of public information at non-occupied patches, the ability to interpret their suitability is necessarily imperfect^11^,^13^. Nearly 50% of the new colonies disappeared a few years (or even a single year) after colonisation^21^. Little evidence was available regarding breeding performance during the first years after colonisation events and it did not show a clear pattern^6^,^20^,^22^.

In summary, we show that breeding experience and longer prospecting periods might be necessary for colonisation. Until now, dispersal theory failed to acknowledge the role of personal information in colonisation for social species^3^,^4^,^9^. Future attempts to understand colonisation in social species should focus on how individuals manage and reduce uncertainty when assessing patch suitability^11^. Population models should account for the higher demographic value of experienced breeders since metapopulation dynamics might be more sensitive to those individuals than previously thought. Our results have significant implications for metapopulation ecology but especially for the understanding of how social species respond to environmental change^1^,^2^.

## Methods

Audouin’s gull is an endemic Mediterranean seabird^5^. Until the mid-2000s, 70% of their world population was concentrated in the Punta de la Banya (source colony, Ebro Delta, 40°37′N, 00°35′E)^9^. However, from 2002 a series of new colonies became established in the western Mediterranean (Fig. 1, Table 1 and electronic Supplementary Material S1). A long-term monitoring and mark-capture-recapture program was established at the source Punta de la Banya colony and has been running since 1981, which allowed us to evaluate possible drivers of colonisations^23^.

### Environmental and breeding performance variables

We used several environmental factors as proxies of adverse breeding conditions: density of aerial nest predators and intra-guild competitors such as the yellow-legged gull *Larus michahellis* (N_Lm_/N_Ia_, yellow-legged gull population size divided by Audouin’s gull population size to account for density-dependence)^24^; presence of a single badger (*Meles meles)* that predated on nests in 1994; regular presence of foxes (*Vulpes vulpes*) preying on nests and adults from 1997 onwards; and extreme weather conditions in 2008 (namely, a strong cold storm that killed most chicks)^13^,^25^,^26^,^27^.

Moreover, we also used Audouin’s gull breeding success as a proxy of adverse breeding conditions. Breeding success was first calculated qualitatively (BSq), categorizing marked individuals as unsuccessful (0, no hatchlings) or successful breeders (1, at least one hatchling); then quantitatively at a population level (BSp) by dividing the number of chicks by colony size (number of pairs). Number of chicks was estimated by capture-mark-recapture using the Lincoln-Petersen estimator and colony size by counting nests using linear transects^5^,^28^,^29^. Breeding success data were available for the source colony and for only one of the newly established colonies (La Ràpita Port).

We considered individual age as a proxy of breeding experience. Most Audouin’s gulls in the source colony recruit at the age of 3 and 4. Therefore, we categorized individuals as inexperienced (3–4 years old) vs. experienced (≥5 years old)^30^. Age of individuals was available for 6 colonies in the year of colonization (Llobregat, Castellón port, La Ràpita port, Tarragona port, St. Antoni and Barcelona port) and for the source colony (Punta de la Banya) (Fig. 1 and Table 1). Since the number of individuals colonising each site was commonly low, we lumped resighting data from all 6 new colonies together and compared their age structure with the age structure at the source colony the same years these new colonies were established (2010, 2011, 2013 and 2014, Fig. 1, Table 1).

Finally, as proxies of breeding performance we recorded the breeding phenology (LD, laying date of the first egg as the number of days elapsed since 1^st^ of April, n = 31), clutch size (CS, the number of eggs laid by clutch, n = 50) and egg volume (V, n = 50). Egg volume (in cm^3^) was calculated using the equation: *V* = β (*L*)(*W*)^2^, in which β was a species-specific constant parameter (β = 0.476 for Audouin’s gull^31^,^32^), *L* was egg length and *W* was egg width, the two expressed in cm. All measures were completed with a digital calliper to the nearest millimetre.

### Data analyses

We first assessed the existence of an association between adverse breeding conditions and colonization events. To do this, we calculated the natural logarithm of the accumulated frequency of new colonies over time and searched for breaking points using Bayesian analysis of change point problems implemented in the “*bcp*” R package^33^. Breaking point analyses detects points that divide data series into blocks such that the mean is constant within each block using the Bayesian statistic framework^34^. We then qualitatively assessed if there was a temporal association between the resulting breaking points and different adverse breeding conditions (badger and fox presence and extreme weather events), density dependence (N_Lm_/N_Ia_) and breeding success (BS).

We assessed if there were differences in the proportions of inexperienced and experienced breeders present between the new and the source colony (n = 827 and 4810 individuals, respectively) by means of a contingency table and a *χ*^2^ test. We tested the hypothesis that individuals having poorer breeding performance the year before were more likely to colonize a new patch than those experiencing high breeding performance in three different ways. First, we used count data, a contingency table and a *χ*^2^ test to compare the proportion of successful and unsuccessful breeders (BSq_t–1_) that, having bred at the source colony in the year previous to the colonization (n = 124), decided to return and breed again at the source colony (n = 102) or to settle in a new colony (n = 22). Second, we used individual data and binomial logistic regression (0 = source colony, 1 = new colony) to test the effect of previous breeding status (successful and unsuccessful) as an explanatory variable for colonization. Third we tested differences in laying date (LD), clutch size (CS) and egg volume (V) in the year previous to the colonization (t-1) in the source colony by individuals present at new (n = 91) and source colonies (n = 109) the year of colonization (t). To test for differences in LD_t–1_ and CS_t-1_ we used linear models, and to test for differences in egg volume (V_t–1_) we used general linear models including nest identity as a random factor (See ref. 35 for details).

We tested the hypothesis that individuals breeding at new colonies should experience higher breeding success by comparing breeding success at the new colony with breeding success at the source colony the year of colonization. At the source colony, breeding individuals are spatially aggregated in discrete dunes and dikes (i.e., patches)—each of these spatial aggregations is considered a sub-colony which usually has a different age distribution of breeding individuals^36^ and habitat characteristics. To eliminate any confounding effect of a different age distribution, we first tested for differences in breeding success with the whole source colony, and then with two sub-colonies (Sub1 called Miseria and Sub2 called Alfacs) within the source colony. These sub-colonies had similar age distributions to the new colony (La Ràpita Port) and differed from one another in their accessibility to terrestrial predators.

Models were selected using the Akaike Information Criterion corrected for overdispersion (AIC_c_^37^). We considered the model with lowest AIC_c_ as the best model, and those within two ΔAIC_c_ (the difference in AIC_c_ values) to be statistically equivalent^37^. All analyses were implemented using the R software.

### Ethics statement

This study complies with the European laws regulating research on animals. Spanish regulation does not require specific ethical approval for wildlife monitoring except from regular permits. Permits were given by Spanish Government: SF/134, SF/043, SF/097 and SF/269.

## Additional Information

**How to cite this article**: Payo-Payo, A. *et al*. Colonisation in social species: the importance of breeding experience for dispersal in overcoming information barriers. *Sci. Rep.* **7**, 42866; doi: 10.1038/srep42866 (2017).

**Publisher's note:** Springer Nature remains neutral with regard to jurisdictional claims in published maps and institutional affiliations.

## Supplementary Material

Supplementary Material

Supplementary Video

## Figures and Tables

**Table 1:** Names of new colonies formed during the study (see Fig. 1 for location), and their characteristics: type of habitat, year of colonization and number of pairs in the establishment year.

Site	Habitat	Year	Pairs
Columbretes	Rocky island	1974	45
Punta de la Banya^*^	Brackish marshes and salt-pans	1981	23
Grosa	Rocky island	1993	300
Albufera	Shallow coastal lagoon	2003	6
El Saler	Artificial coastal lagoon	2004	1
Torrevieja	Salt-pans	2005	30
Benidorm	Rocky island	2006	3
Tomás Maestre	Port	2006	11
San Pedro	Salt-pans	2006	18
Almenara	Shallow coastal lagoon	2009	5
Llobregat^*^	Artificial riverine island	2010	140
Escombreras	Port	2010	3
Buda	Brackish marshes	2011	1
Castellón^*^	Port	2011	303
La Ràpita^*^	Port	2011	2609
Sollana	Shallow coastal lagoon	2012	1
Tarragona^*^	Port	2013	19
Barcelona^*^	Port	2013	69
Sant Antoni^*^	Brackish marshes and salt-pans	2014	116
Valencia	Port	2014	239

New colonies are sorted by year of colonization, except for Columbretes Is, which was likely settled before. Punta de la Banya is the source colony^9^.

^*^Colonies used to assess age structure.
