# Supplementary material for: Colonisation in social species: the importance of breeding experience for dispersal in overcoming information barriers
Source: Sci Rep. 2017 Feb 17;7:42866. doi: 10.1038/srep42866 (PMC5314353; doi:10.1038/srep42866)
Supplement: Supplementary Material [file srep42866-s1.pdf]

# Electronic supplementary material

Colonisation in social species: the importance of breeding experience for dispersal in overcoming information barriers

Payo-Payo, A.; Genovart, M.; Sanz-Aguilar, A.; Greño, J.; García-Tarasón, M; Bertolero, A.; Piccardo, J.; Oro, D.

**Electronic supplementary material S1:**

Video 1: Temporal evolution of Audouin's gull breeding colonies along the western Mediterranean coast at regular time intervals between 1983 and 2014. Circle size is proportional to the number of colonizers in the year of colony foundation; anchors indicate colonies settled in port areas (See Table 1 for colony details). Maps were built in R-Software [38]

Video_colonisation_in_social_species.mp4

**Electronic supplementary material S2:**

Table 2. Table summarizing the model selection for previous year breeding status of Audouin's gulls breeding in the Western Mediterranean coast. $COL_t$: colony in time t (source/new); CST, constant; $BS_{t-1}$, previous season breeding status; DEV, deviance; AICc: corrected Akaike's information criterion; ΔAICc: AICc difference with the best model.

| | Model | Deviance | AICc | ΔAICc |
|---|---|---|---|---|
| $COL_t$ | CST | | 117 | 0 |
| $COL_t$ | $BS_{t\ 1}$ | -0.725 | 118.2 | 1.2 |

Table 3. Table summarizing the model selection for previous year breeding performance of Audouin's gulls breeding in the Western Mediterranean coast. $V_{t-1}$ , egg volume, $CS_{t-1}$, clutch size; $LD_{t-1}$ laying date; CST, constant, COLONY, source or new colony; DEV, deviance; AICc: corrected Akaike's information criterion; ΔAICc: AICc difference with the best model.*Includes nest as a random effect.

| | Model | Deviance | AICc | ΔAICc |
|---|---|---|---|---|
| $V_{t-1}$ | CST* | 252 | 258 | 2 |
| $V_{t-1}$ | COLONY* | 250 | 256 | 0 |
| | | | | |
| $CS_{t-1}$ | CST | 30 | 123 | 0 |
| $CS_{t-1}$ | COLONY | 30 | 125 | 2 |
| | | | | |
| $LD_{t-1}$ | CST | 1506 | 212 | 0 |
| $LD_{t-1}$ | COLONY | 1478 | 214 | 2 |
